# *N*,*N*′-Bis(2-chloro­phen­yl)succinamide

**DOI:** 10.1107/S1600536811013407

**Published:** 2011-04-16

**Authors:** B. S. Saraswathi, Sabine Foro, B. Thimme Gowda

**Affiliations:** a Department of Chemistry, Mangalore University, Mangalagangotri 574 199, Mangalore, India; b Institute of Materials Science, Darmstadt University of Technology, Petersenstrasse 23, D-64287 Darmstadt, Germany

## Abstract

There is one half-mol­ecule in the asymmetric unit of the title compound, C_16_H_14_Cl_2_N_2_O_2_, with a center of symmetry at the mid-point of the central C—C bond. The N—H and C=O bonds in the C—NH—C(O)—C fragment are *anti* to each other and the amide O atom is *anti* to the H atoms attached to the adjacent C atoms. However, the conformation of the N—H bond in the amide fragments is *syn* to the *ortho*-chloro groups in the adjacent benzene rings. The dihedral angle between the benzene ring and the NH—C(O)—CH_2_ fragment is 47.0 (2)°. In the crystal, a series of N—H⋯O inter­molecular hydrogen bonds link the mol­ecules into chains along the *b* axis.

## Related literature

For our study of the effect of substituents on the structures of *N*-(ar­yl)-amides, see: Gowda *et al.* (2000 ▶); Saraswathi *et al.* (2011**a* ▶,b* ▶) and on *N*-(ar­yl)-methane­sulfonamides, see: Gowda *et al.* (2007 ▶). For a similar structure, see Pierrot *et al.* (1984 ▶).
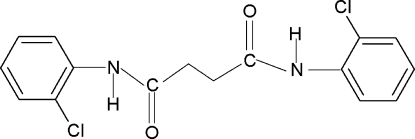


## Experimental

### 

#### Crystal data

C_16_H_14_Cl_2_N_2_O_2_
*M* *_r_* = 337.19
Monoclinic, 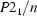

*a* = 4.820 (2) Å
*b* = 11.445 (3) Å
*c* = 14.242 (4) Å
β = 98.10 (3)°
*V* = 777.8 (4) Å^3^
*Z* = 2
Mo *K*α radiation
μ = 0.43 mm^−1^
*T* = 293 K
0.44 × 0.08 × 0.04 mm

#### Data collection

Oxford Diffraction Xcalibur diffractometer with a Sapphire CCD detector
Absorption correction: multi-scan (*CrysAlis RED*; Oxford Diffraction, 2009 ▶) *T*_min_ = 0.835, *T*_max_ = 0.983
2501 measured reflections
1563 independent reflections
900 reflections with *I* > 2σ(*I*)
*R*_int_ = 0.038

#### Refinement

*R*[*F* ^2^ > 2σ(*F* ^2^)] = 0.075
*wR*(*F* ^2^) = 0.139
*S* = 1.16
1563 reflections
103 parameters
1 restraint
H atoms treated by a mixture of independent and constrained refinement
Δρ_max_ = 0.25 e Å^−3^
Δρ_min_ = −0.22 e Å^−3^

### 

Data collection: *CrysAlis CCD* (Oxford Diffraction, 2009 ▶); cell refinement: *CrysAlis RED* (Oxford Diffraction, 2009 ▶); data reduction: *CrysAlis RED*; program(s) used to solve structure: *SHELXS97* (Sheldrick, 2008 ▶); program(s) used to refine structure: *SHELXL97* (Sheldrick, 2008 ▶); molecular graphics: *PLATON* (Spek, 2009 ▶); software used to prepare material for publication: *SHELXL97*.

## Supplementary Material

Crystal structure: contains datablocks I, global. DOI: 10.1107/S1600536811013407/fl2341sup1.cif

Structure factors: contains datablocks I. DOI: 10.1107/S1600536811013407/fl2341Isup2.hkl

Additional supplementary materials:  crystallographic information; 3D view; checkCIF report

## Figures and Tables

**Table 1:** Hydrogen-bond geometry (Å, °)

*D*—H⋯*A*	*D*—H	H⋯*A*	*D*⋯*A*	*D*—H⋯*A*
N1—H1*N*⋯O1^i^	0.86 (2)	2.11 (2)	2.936 (4)	161 (3)

Symmetry code: (i) 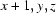.

**Fig. 1.**
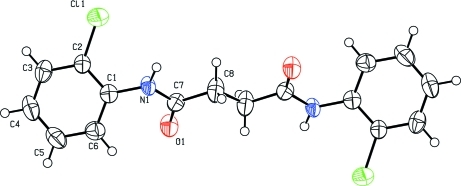
Molecular structure of (I), showing the atom labelling scheme and displacement ellipsoids are drawn at the 50% probability level.

**Fig. 2.**
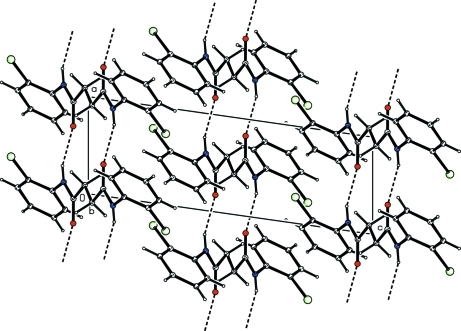
Molecular packing of (I) with hydrogen bonding shown as dashed lines.

## supplementary crystallographic information

### Comment

Amide and sulfonamide moieties are important constituents of many biologically significant compounds. As a part of studying the substituent effects on structures of this class of compounds(Gowda *et al.*, 2000, 2007; Saraswathi *et al.*, 2011**a*,b*), the structure of (I) has been determined (Fig.1). (I) sits on a center of symmetry passing through the mid-point of the central C—C bond to give a half molecule per asymmetric unit. This is similar to that obseved in bis(2-chlorophenylaminocarbonylmethyl)disulfide (II)(Pierrot *et al.*, 1984), *N*,*N*-bis(2-methylphenyl)-succinamide (III)(Saraswathi *et al.*, 2011*a*) and *N*,*N*-bis(3-chlorophenyl)- succinamide (III)(Saraswathi *et al.*, 2011*b*).

The conformations of the N—H and C=O bonds in the C—NH—C(O)—C segments are *anti* to each other and the amide O atoms are *anti* to the H atoms attached to the adjacent C atoms. But the conformations of the N—H bonds in the amide fragments are *syn* to the *ortho*- chloro groups in the adjacent benzene rings, in contrast to the *anti* conformations observed with respect to the *ortho*-methyl groups in (III) and with respect to the *meta*-chloro groups in (IV).

The dihedral angle between the benzene ring and the NH—C(O)—CH_2_ segment in the two halves of the molecule is 47.0 (2)°, compared to the values of 62.1 (2)° in (III) and 32.8 (1)° in (Iv).

The torsion angles of N1–C7–C8–C8a and O1–C7–C8–C8a in (I) are 172.2 (5)° and -7.8 (4)°, in contrast to the values of 150.9 (3)° and -30.5 (4)° in (III) and -175.4 (2)° and 5.9 (4)° in (IV). The differences in the torsion angles may be due to the steric hindrances caused by the different substituents.

Similarly, the torsion angles of C2—C1—N1—C7 and C6—C1—N1—C7 are -47.6 (6)° and 133.7 (4)°, compared to the values of -64.0 (4)° and 117.6 (3)° in (III) and -35.0 (3)° and 147.5 (2)° in (IV).

The packing of molecules in the crystal linked by of N—H···O hydrogen bonds (Table 1) is shown in Fig. 2.

### Experimental

Succinic anhydride (0.01 mol) in toluene (25 ml) was treated drop wise with 2-chloroaniline (0.01 mol) also in toluene (20 ml) with constant stirring. The resulting mixture was stirred for one hour and set aside for an additional hour at room temperature for completion of the reaction. The mixture was then treated with dilute hydrochloric acid to remove unreacted 2-chloroaniline. The resultant solid *N*-(2-chlorophenyl)-succinamic acid was filtered under suction and washed thoroughly with water to remove the unreacted succinic anhydride and succinic acid. The compound was recrystallized to constant melting point from ethanol. The purity of the compound was checked by elemental analysis and characterized by its infrared and NMR spectra.

The *N*-(2-chlorophenyl)succinamic acid obtained was then treated with phosphorous oxychloride and excess of 2-chloroaniline at room temperature with constant stirring. The resultant mixture was stirred for 4 h, kept aside for additional 6 h for completion of the reaction and poured slowly into crushed ice with constant stirring. It was kept aside for a day. The resultant solid, *N*,*N*-bis(2-chlorophenyl)- succinamide was filtered under suction, washed thoroughly with water, dilute sodium hydroxide solution and finally with water. It was recrystallized to constant melting point from a mixture of acetone and chloroform. The purity of the compound was checked by elemental analysis, and characterized by its infrared and NMR spectra.

Needle like colorless single crystals used in the X-ray diffraction studies were were grown in a mixture of acetone and chloroform at room temperature.

### Refinement

The H atom of the NH group was located in a difference map and later restrained to the distance N—H = 0.86 (2) Å. The other H atoms were positioned with idealized geometry using a riding model with the aromatic C—H = 0.93Å and the methylene C—H = 0.97 Å. All H atoms were refined with isotropic displacement parameters (set to 1.2 times of the *U*_eq_ of the parent atom).

### Crystal data

**Table:**

C_16_H_14_Cl_2_N_2_O_2_	*F*(000) = 348
*M**_r_* = 337.19	*D*_x_ = 1.440 Mg m^−^^3^
Monoclinic, *P*2_1_/*n*	Mo *K*α radiation, λ = 0.71073 Å
Hall symbol: -P 2yn	Cell parameters from 475 reflections
*a* = 4.820 (2) Å	θ = 2.9–27.8°
*b* = 11.445 (3) Å	µ = 0.43 mm^−^^1^
*c* = 14.242 (4) Å	*T* = 293 K
β = 98.10 (3)°	Needle, colourless
*V* = 777.8 (4) Å^3^	0.44 × 0.08 × 0.04 mm
*Z* = 2	

### Data collection

**Table:**

Oxford Diffraction Xcalibur diffractometer with a Sapphire CCD detector	1563 independent reflections
Radiation source: fine-focus sealed tube	900 reflections with *I* > 2σ(*I*)
graphite	*R*_int_ = 0.038
Rotation method data acquisition using ω scans	θ_max_ = 26.4°, θ_min_ = 2.9°
Absorption correction: multi-scan (*CrysAlis RED*; Oxford Diffraction, 2009)	*h* = −6→5
*T*_min_ = 0.835, *T*_max_ = 0.983	*k* = −10→14
2501 measured reflections	*l* = −17→6

### Refinement

**Table:**

Refinement on *F*^2^	Primary atom site location: structure-invariant direct methods
Least-squares matrix: full	Secondary atom site location: difference Fourier map
*R*[*F*^2^ > 2σ(*F*^2^)] = 0.075	Hydrogen site location: inferred from neighbouring sites
*wR*(*F*^2^) = 0.139	H atoms treated by a mixture of independent and constrained refinement
*S* = 1.16	*w* = 1/[σ^2^(*F*_o_^2^) + (0.0376*P*)^2^ + 0.5594*P*] where *P* = (*F*_o_^2^ + 2*F*_c_^2^)/3
1563 reflections	(Δ/σ)_max_ = 0.002
103 parameters	Δρ_max_ = 0.25 e Å^−^^3^
1 restraint	Δρ_min_ = −0.22 e Å^−^^3^

### Special details

**Table:**

Experimental. CrysAlis RED (Oxford Diffraction, 2009) Empirical absorption correction using spherical harmonics, implemented in SCALE3 ABSPACK scaling algorithm.
Geometry. All e.s.d.'s (except the e.s.d. in the dihedral angle between two l.s. planes) are estimated using the full covariance matrix. The cell e.s.d.'s are taken into account individually in the estimation of e.s.d.'s in distances, angles and torsion angles; correlations between e.s.d.'s in cell parameters are only used when they are defined by crystal symmetry. An approximate (isotropic) treatment of cell e.s.d.'s is used for estimating e.s.d.'s involving l.s. planes.
Refinement. Refinement of *F*^2^ against ALL reflections. The weighted *R*-factor *wR* and goodness of fit *S* are based on *F*^2^, conventional *R*-factors *R* are based on *F*, with *F* set to zero for negative *F*^2^. The threshold expression of *F*^2^ > σ(*F*^2^) is used only for calculating *R*-factors(gt) *etc*. and is not relevant to the choice of reflections for refinement. *R*-factors based on *F*^2^ are statistically about twice as large as those based on *F*, and *R*- factors based on ALL data will be even larger.

### Fractional atomic coordinates and isotropic or equivalent isotropic displacement parameters (Å^2^)

**Table:**

	*x*	*y*	*z*	*U*_iso_*/*U*_eq_	
C1	−0.0199 (7)	0.1565 (3)	0.8739 (3)	0.0360 (10)	
C2	0.0563 (7)	0.1162 (3)	0.7889 (3)	0.0372 (10)	
C3	−0.0371 (9)	0.0099 (4)	0.7516 (3)	0.0486 (11)	
H3	0.0193	−0.0164	0.6955	0.058*	
C4	−0.2122 (9)	−0.0569 (4)	0.7966 (4)	0.0584 (13)	
H4	−0.2760	−0.1283	0.7710	0.070*	
C5	−0.2940 (10)	−0.0188 (4)	0.8796 (4)	0.0596 (13)	
H5	−0.4151	−0.0639	0.9099	0.072*	
C6	−0.1964 (9)	0.0869 (4)	0.9185 (3)	0.0490 (11)	
H6	−0.2504	0.1114	0.9755	0.059*	
C7	−0.0733 (8)	0.3461 (4)	0.9476 (3)	0.0392 (10)	
C8	0.0844 (9)	0.4520 (4)	0.9871 (4)	0.0621 (14)	
H8A	0.2115	0.4287	1.0430	0.075*	
H8B	0.1977	0.4799	0.9406	0.075*	
N1	0.0847 (6)	0.2633 (3)	0.9133 (2)	0.0392 (9)	
H1N	0.251 (5)	0.287 (3)	0.909 (3)	0.047*	
O1	−0.3255 (5)	0.3362 (2)	0.9469 (2)	0.0506 (9)	
Cl1	0.2688 (2)	0.20113 (11)	0.72809 (8)	0.0586 (4)	

### Atomic displacement parameters (Å^2^)

**Table:**

	*U*^11^	*U*^22^	*U*^33^	*U*^12^	*U*^13^	*U*^23^
C1	0.0217 (18)	0.040 (2)	0.045 (2)	0.0046 (18)	−0.0007 (18)	−0.002 (2)
C2	0.028 (2)	0.037 (2)	0.046 (3)	0.0015 (19)	0.0026 (18)	−0.006 (2)
C3	0.041 (2)	0.050 (3)	0.052 (3)	0.004 (2)	−0.002 (2)	−0.016 (2)
C4	0.046 (3)	0.039 (3)	0.088 (4)	−0.006 (2)	0.002 (3)	−0.014 (3)
C5	0.052 (3)	0.047 (3)	0.081 (4)	−0.013 (2)	0.012 (3)	0.002 (3)
C6	0.043 (3)	0.051 (3)	0.054 (3)	−0.002 (2)	0.012 (2)	−0.001 (2)
C7	0.025 (2)	0.049 (3)	0.043 (2)	−0.0019 (19)	0.0035 (18)	−0.010 (2)
C8	0.030 (2)	0.061 (3)	0.096 (4)	−0.005 (2)	0.013 (2)	−0.040 (3)
N1	0.0212 (17)	0.035 (2)	0.063 (2)	−0.0055 (16)	0.0102 (17)	−0.0159 (17)
O1	0.0202 (14)	0.0527 (19)	0.079 (2)	−0.0032 (13)	0.0082 (14)	−0.0217 (16)
Cl1	0.0566 (7)	0.0597 (7)	0.0648 (8)	−0.0032 (7)	0.0266 (6)	−0.0035 (7)

### Geometric parameters (Å, °)

**Table:**

C1—C6	1.384 (5)	C5—H5	0.9300
C1—C2	1.393 (5)	C6—H6	0.9300
C1—N1	1.408 (5)	C7—O1	1.220 (4)
C2—C3	1.377 (5)	C7—N1	1.350 (5)
C2—Cl1	1.730 (4)	C7—C8	1.498 (5)
C3—C4	1.364 (6)	C8—C8^i^	1.445 (8)
C3—H3	0.9300	C8—H8A	0.9700
C4—C5	1.369 (6)	C8—H8B	0.9700
C4—H4	0.9300	N1—H1N	0.857 (19)
C5—C6	1.384 (6)		
			
C6—C1—C2	117.6 (4)	C5—C6—C1	121.0 (4)
C6—C1—N1	121.7 (4)	C5—C6—H6	119.5
C2—C1—N1	120.7 (4)	C1—C6—H6	119.5
C3—C2—C1	121.1 (4)	O1—C7—N1	123.0 (4)
C3—C2—Cl1	119.2 (3)	O1—C7—C8	122.1 (4)
C1—C2—Cl1	119.7 (3)	N1—C7—C8	115.0 (3)
C4—C3—C2	120.3 (4)	C8^i^—C8—C7	115.9 (4)
C4—C3—H3	119.9	C8^i^—C8—H8A	108.3
C2—C3—H3	119.9	C7—C8—H8A	108.3
C3—C4—C5	120.0 (4)	C8^i^—C8—H8B	108.3
C3—C4—H4	120.0	C7—C8—H8B	108.3
C5—C4—H4	120.0	H8A—C8—H8B	107.4
C4—C5—C6	120.1 (4)	C7—N1—C1	124.4 (3)
C4—C5—H5	120.0	C7—N1—H1N	113 (3)
C6—C5—H5	120.0	C1—N1—H1N	122 (3)
			
C6—C1—C2—C3	−1.1 (6)	C2—C1—C6—C5	−0.2 (6)
N1—C1—C2—C3	177.7 (4)	N1—C1—C6—C5	−178.9 (4)
C6—C1—C2—Cl1	178.3 (3)	O1—C7—C8—C8^i^	−7.8 (9)
N1—C1—C2—Cl1	−3.0 (5)	N1—C7—C8—C8^i^	172.2 (5)
C1—C2—C3—C4	1.4 (6)	O1—C7—N1—C1	−0.6 (7)
Cl1—C2—C3—C4	−177.9 (3)	C8—C7—N1—C1	179.3 (4)
C2—C3—C4—C5	−0.5 (7)	C6—C1—N1—C7	−47.6 (6)
C3—C4—C5—C6	−0.8 (7)	C2—C1—N1—C7	133.7 (4)
C4—C5—C6—C1	1.2 (7)		

Symmetry codes: (i) −*x*, −*y*+1, −*z*+2.

### Hydrogen-bond geometry (Å, °)

**Table:**

*D*—H···*A*	*D*—H	H···*A*	*D*···*A*	*D*—H···*A*
N1—H1N···O1^ii^	0.86 (2)	2.11 (2)	2.936 (4)	161 (3)

Symmetry codes: (ii) *x*+1, *y*, *z*.
